# Albaconazole Polymeric Nanocapsules for Treating *Trypanosoma cruzi* Infections

**DOI:** 10.3390/pathogens14040319

**Published:** 2025-03-26

**Authors:** Cristina Maria de Barros, Vanja Maria Veloso, Margareth Spangler Andrade, José Mário Carneiro Vilela, Maria Alice de Oliveira, Marta de Lana, Maria Terezinha Bahia, Vanessa Carla Furtado Mosqueira

**Affiliations:** 1 Laboratory of Pharmaceutics and Nanotechnology, School of Pharmacy, Federal University of Ouro Preto, Campus Universitário Morro do Cruzeiro, Ouro Preto 35400-000, MG, Brazil; 2 Post-Graduation Program in Biological Sciences (NUPEB), Exact and Biological Sciences Institute (ICEB), Federal University of Ouro Preto, Ouro Preto 35400-000, MG, Brazil; 3 Department of Pharmacy, School of Pharmacy, Federal University of Ouro Preto, Ouro Preto 35400-000, MG, Brazil; 4 Laboratory of Nanoscopy, FIEMG SENAI Technological Center—Regional Department of Minas Gerais, Belo Horizonte 31035-536, MG, Brazil; 5 Department of Clinical Analysis, School of Pharmacy, Federal University of Ouro Preto, Ouro Preto 35400-000, MG, Brazil; 6 Parasitic Diseases Laboratory, School of Medicine, Federal University of Ouro Preto, Ouro Preto 35400-000, MG, Brazil

**Keywords:** albaconazole, UR-9825, nanoparticles, efficacy, toxicity, formulation, dose–response, dosage regimen

## Abstract

The therapeutic management of Chagas disease requires new medicines because the standard-of-care drugs available induce adverse effects and have limited efficacy. In this study, we developed a formulation of albaconazole (ABZ) loaded in biodegradable polymeric nanocapsules (NCs). Free ABZ and ABZ-loaded NCs were similarly active against the Y strain and inactive against the Colombian strain epimastigotes of *Trypanosoma cruzi*. Infected mice were given ABZ in different doses and treatment schedules by oral, SC, and IM routes during the acute phase of infection. Free ABZ taken orally reduced parasitemia and suppressed mortality; however, all the animals maintained patent parasitemia during and after treatment. ABZ-NCs increased anti-*T. cruzi* effects (*p* < 0.05), inducing negative parasitemia during treatment in most of the tested regimens. The parasitemia level was also significantly reduced after treatment with ABZ-NCs during the acute phase of the disease, and relapses were delayed compared with the free ABZ treatment. Once- and twice-daily doses were similarly effective, demonstrating that the NCs prolonged the ABZ-NC residence time. Free ABZ and ABZ-NCs did not prevent infection, ABZ seemed to have suppressive effects on *T. cruzi* growth, and encapsulation prolonged this suppression. The analysis of the in vivo results indicated that the NCs significantly improved the safety of ABZ in the mouse model, suggesting that the increased ABZ-NC dosage regimen merits further efficacy and pharmacokinetic evaluations.

## 1. Introduction

Chagas disease (American Trypanosomiasis) is caused by the protozoan *Trypanosoma cruzi*. It is estimated that approximately 6 million individuals have chronic infection with this pathogen, mostly in Latin America [1,2]. Chagas disease (CD) has a significant social impact in Latin America and is a neglected tropical disease [3]. In CD endemic areas, the only drug available for treatment is benznidazole (BZN). However, treatment with BZN has serious side effects in addition to low cure rates during the chronic phase of the infection [2,4]. In clinical trials performed during the acute phase of the infection, the cure rate obtained was 40% to 76% [5]. Long-term studies on chemotherapy with BZN for children and adolescents with short-term chronic infections have also shown significant success [6,7]. In different regions of Bolivia, however, BZN treatment in children and adolescents induced cure rates of only 0% to 5.4% [8]. No effective treatment exists for the established chronic form of the disease, which is currently the most common clinical presentation in both endemic and non-endemic areas [5,9].

New active pharmaceutical ingredients (APIs) with low toxicity and high cure rates in the acute and chronic phases of CD are urgently needed to control this infection, which is spreading in various countries and continents [1,2,4,10]. A new class of synthetic azole molecules with antifungal activity, such as posaconazole, albaconazole (ABZ), voriconazole, TAK-187, and D0870, has been investigated as an alternative approach to chemotherapy in Chagas disease, because they act as selective inhibitors of lanosterol C-14α-demethylase (CYP51 or Erg11p), a key enzyme implicated in ergosterol synthesis [10,11,12,13]. This is a common pathway for the fungi and protozoa of the Trypanosomatidae family, including *T. cruzi* [14]. Triazoles have variable affinity for CYP51, and their action as CYP51 inhibitors depletes ergosterol, causing methylated sterols to accumulate in the cell membrane, which disrupts membrane fluidity, inhibits growth, or induces cell death [14].

**Figure 1:**
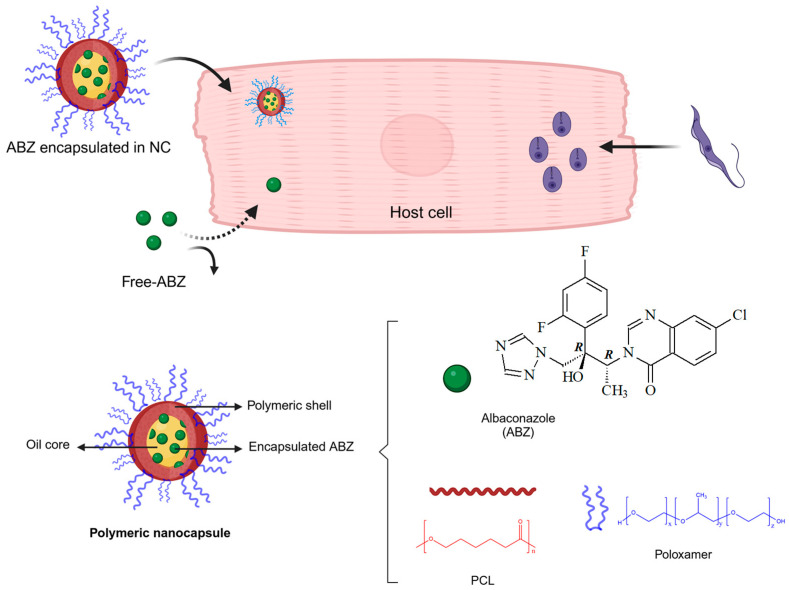
Schematic representation of nanocapsule structure, the chemical structure of ABZ, and the strategy used to deliver ABZ inside *T. cruzi*-infected host cells. Created in BioRender’s web-based software 2025. https://BioRender.com/i26s954 (accessed on 21 February 2025).

Among the azole candidates, ABZ (UR-9825, Group Uriach) [12,13,14,15,16] is a triazole antifungal with a broad spectrum of activity and high oral bioavailability in humans [17,18,19]. ABZ’s chemical structure is shown in Figure 1. Apart from its potent effects against life-threatening and invasive fungal infections [20,21], this drug is also active against *T. cruzi* in vitro and in vivo [22,23].

However, ABZ has solubility limitations. It is a lipophilic molecule (clog *P*_o/w_ 2.56) and has a short plasma half-life in mice (<1 h) [12,13,15]. Thus, the development of ABZ as an anti-*T. cruzi* candidate is hampered, since it is challenging to evaluate its efficacy in a preclinical murine experimental model of *T. cruzi* infection due to its fast elimination and the inability to detect it in plasma 6 h post-administration [12]. ABZ efficacy has been investigated in dogs infected with the Y and Berenice-78 strains of *T. cruzi*, where it showed promising results in suppressing parasitemia and increasing survival. Cure rates of 25% and 100% were obtained among animals infected with strain Y and treated with 1.5 mg/kg/day of ABZ for 60 and 90 days, respectively [23]. Furthermore, repeated toxicity studies in rats for 28 days with 100 mg/kg twice a day and 250 mg/kg once a day showed a low incidence of toxic effects [24,25].

More recently, ABZ was evaluated in clinical trials (phase II/III), namely, NCT00199264, NCT00509275, and NCT00730405, as an antifungal [17,18,19,24,26,27,28]. These studies revealed that ABZ has good pharmacokinetic and oral bioavailability profiles for a once-a-week dosing schedule during 9 months of treatment to control mycoses in humans [19]. ABZ’s antifungal effects were dose-dependent [19]. In this phase II clinical trial testing different oral doses (100, 200, 300, and 400 mg/kg), ABZ’s toxicological profile showed that it was prone to causing gastrointestinal events such as nausea and diarrhea. However, it was well tolerated, with mild–moderate adverse effects occurring in less than 3% of patients [19].

ABZ has already been formulated as tablets and capsules, and clinical trials comparing these dosage forms demonstrated different bioavailabilities [29]. Its poor water solubility and slow dissolution rate impaired the development of parenteral formulations, and, for this reason, there are no intravenous and subcutaneous injectable formulations of this drug.

Nanocapsules (NCs) represent a dosage form that has demonstrated outstanding therapeutic properties by reducing the toxic effects of anti-*T. cruzi* drugs [30], modifying biodistribution [31], and improving efficacy [32]. This delivery system has been used for encapsulating different lipophilic drugs [33]. We recently reported that NCs effectively interact with infected host cells [34]. After being internalized by them, NCs can deliver the encapsulated drug to the vicinity of intracellular *T. cruzi* amastigotes. NCs have also been shown to interact with blood trypomastigotes [34].

The association of ABZ with polymeric biodegradable NCs is envisaged in the present study, aiming to provide prolonged release, as well as circumventing toxicity issues related to azole derivatives, such as QTc prolongation and arrhythmia [35,36,37]. NCs have been shown to be effective in reducing both in vitro and in vivo drug-induced cardiotoxicity [38,39]. These expected NC properties may offer relief for patients in the chronic phase of Chagas disease by potentially reducing adverse effects. Furthermore, parenteral (SC, IV and IM) dosage forms for treating *T. cruzi* infections are not currently available. NCs can be administered by SC, IM, IV, and oral routes. Another possible useful application for this delivery system would be to control serious cases, such as those caused by oral infections mediated by contaminated food [40].

The present study reports the development of biodegradable ABZ-loaded NCs, chosen due to the ability of their oily core to entrap lipophilic compounds (Figure 1). The pre-clinical pharmaceutical development and the physicochemical characterization of ABZ-NCs, in terms of surface charge, hydrodynamic diameter, polydispersity, drug loading, and encapsulation yield, are also reported in this study. A morphological analysis of these new formulations was also conducted by atomic force microscopy (AFM) and scanning electron microscopy (SEM). These techniques were able to image NCs and their surface features, supplying high-resolution information in nanoscale dimensions. An in vitro biological evaluation using acellular cultures of *T. cruzi* epimastigotes was carried out. Furthermore, the dose-response curve of the parasitemia in mice infected with the Y strain of *T. cruzi* (Y strain) and treated with ABZ-NCs or free ABZ was also investigated using a murine model. We aimed to obtain a slow release of ABZ into the tissues to reduce the frequency of administration and the number of doses. The in vivo toxicity induced by scaling doses of ABZ has also been investigated following IV administration.

## 2. Materials and Methods

### 2.1. Materials

Lecithin, the soy phosphatidylcholine ~70% purity (Epikuron^®^170), was acquired from Lucas Meyer (Hamburg, Germany), and poloxamer 188 MW 8400 g/mol (Synperonic PE/F68) was provided by ICI Surfactants (Cleveland, UK). Poly-ε-caprolactone polymer (PCL; Mn 42,500 g/mol, Ð 1.529 at 25 °C) was purchased from Sigma (Sigma-Aldrich Co., St. Louis, MO, USA). Benznidazole (2-nitroimidazol-(N-benzyl-2-nitro-1-imidazolacetamida) was provided by Roche S.A. (Buenos aires, BAL, Argentina). Medium-chain triglycerides (MCTs) (Miglyol 810N) were a donation from IOI OleoChemical (GmbH, Hamburg, Germany). ABZ (UR-9825), the (1*R*,2*R*)-7-chloro-3-[2-(2,4-difluorophenyl)-2-hydroxy-1-methyl-3-(1H-1,2,4-triazol-1-yl)propyl]quinazolin-4(3H)-one, were kindly given by Javier Bartrolí (Uriach & Cia, Barcelona, Spain) to Prof. J. Urbina (Centro de Biofísica y Bioquímica, Miranda state, Venezuela) and Prof. M.T. Bahia. All other solvents (acetone, *N*,*N*-dimethyl-acetamide, dimethyl sulfoxide, glucose, methanol, and polyethylene glycol 300) were analytical-grade substances and used without further purification. The water was purified by reverse osmosis using the Symplicity^®^ System 185 device (Millipore, Burlington, MA, USA).

### 2.2. Preparation of ABZ Nanocapsules and Solutions

**Figure 2:**
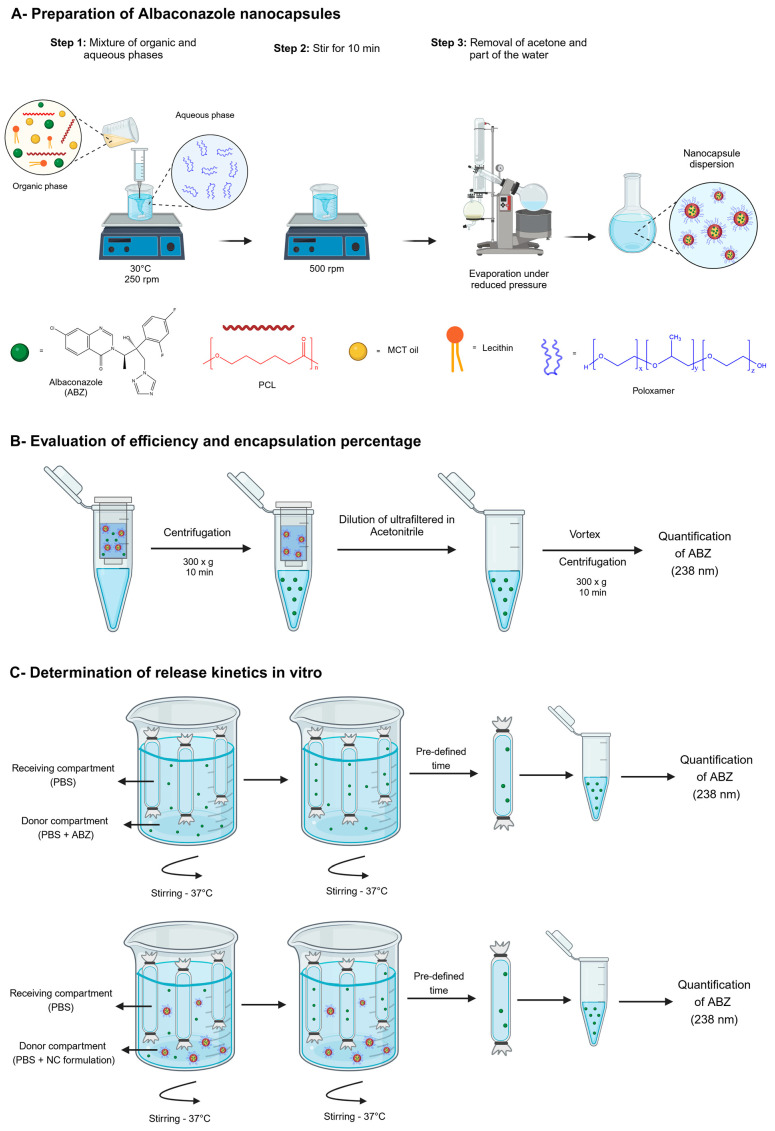
Schematic representation of the preparation method for nanocapsules (**A**); the ultrafiltration/centrifugation method used to separate ABZ loaded in nanocapsules from unloaded ABZ (**B**); and the method of reverse dialysis (**C**) used to study the dissolution/release rate of NCs.

The interfacial deposition method for a preformed polymer followed by the removal of the solvent (nanoprecipitation method) described by Fessi [41] was used to prepare the NCs. The organic phase consisted of 0.6% (*wt*/*v*) polycaprolactone, 0.75% (*wt*/*v*) lecithin, 2.5% (*v*/*v*) MCT oil, and different concentrations of ABZ (0.5, 1.0, and 5.0 mg/mL). The components of the organic phase were dissolved in 10 mL of acetone under magnetic stirring (model PC-200, Corning, NY, USA) at 30 °C at 250 rpm. The organic solution was transferred into the aqueous phase containing 0.75% *wt/v* of poloxamer 188 using a syringe. The mixture was then maintained for 10 min under magnetic stirring (500 rpm). Finally, the colloidal dispersion obtained was concentrated using a rotavapor Laborota 4000/4001 Heidolph (Heidolph Instruments GmbH, Schwabach, Germany), and its final volume was reduced to 10 mL after the complete removal of acetone and part of the water under reduced pressure. Blank NCs, without ABZ, was produced by the same procedure. The preparation process is schematically represented in Figure 2A.

A free ABZ solution was prepared at 2.5 mg/mL. ABZ was dissolved in a mixture composed of (1:2:7) parts of *N*,*N*-dimethyl-acetamide (DMA), PEG 300, and glucose (5% *wt*/*v*). ABZ was first dissolved in DMA, and, afterwards, PEG 300 was added to the solution. The isotonic glucose solution was slowly incorporated into the DMA/PEG 300 solution under low agitation (MS1 vortex apparatus, IKA Works, Campinas, SP, Brazil).

### 2.3. ABZ Quantification by Ultraviolet Spectrometry

An ultraviolet (UV)-based methodology was developed and validated to quantify free ABZ and ABZ loaded in NCs (Supplementary Materials). The ABZ UV–visible spectrum (Helios α, ThermoSpectronic UV-VIS Spectrophotometer, Pittsburgh, PA, USA) was obtained (Figure S1, Supplementary Materials), and the wavelength of 238 nm was selected to measure ABZ concentrations (details in Supplementary Materials). The ABZ calibration curve (standard curve) was built, containing eight points of known ABZ concentrations in acetonitrile (0.25; 0.5; 1.0; 2.5; 5.0; 7.5; 10.0, and 25 µg/mL). The selectivity, specificity, matrix effect (presence of NCs at 1 and 10%), linearity, repeatability, precision, and accuracy were determined according to the acceptance criteria established by the Analytical and Bioanalytical Methods Validation Guide (ANVISA/MS, BRAZIL, 2003) [42]. The method was linear in the range of 0.25 to 25 mg/mL (r^2^ = 0.9986 and equation y = 0.0972x + 0.0226) and was shown to be selective, precise, and accurate in quantifying free ABZ and ABZ in the presence of NC excipients. The method’s intra- and inter-day coefficient of variation were within the maximum established limits. The method was, therefore, considered validated for determining ABZ, and it was applied to quantify ABZ loading and encapsulation efficiency, as well as to quantifying the in vitro release of ABZ from the NCs. Detailed information about this method’s development and validation is given in the Supplementary Material Figures and Tables.

### 2.4. Polymeric Nanocapsule Characterization

The NCs’ mean hydrodynamic diameter (Dh) and polydispersity index (PDI) were determined by dynamic light scattering (DLS) at 25 °C with an angle of 90°. NCs were first diluted in ultrapure water. The zeta potential was determined by electrophoretic light scattering in suitable cuvettes, with samples diluted 1:500 times in 1 mM NaCl solution (f(Ka) = 1.5). Both analyses were conducted using a Zetasizer 3000HS Advanced (Malvern Instruments, Worcestershire, UK). The values presented in the tables and graphs correspond to the mean and standard deviations of a minimum of 3 different batches of NCs with 10 readings per sample, determined in triplicate.

### 2.5. Atomic Force Microscopy and Scanning Electron Microscopy Analysis

The morphological examination of the different nanoparticle formulations was carried out using two different techniques. The SEM images were obtained on a JEOL JSM 5510 microscope at 20 kV, after covering with gold for the shadowing of the samples using sputter equipment. The AFM images were collected using a Dimension 3000 device, monitored by the Nanoscope IIIa controller (Digital Instruments, Santa Barbara, CA, USA), using silicon probes of length 228 μm, with a resonance frequency of 75–98 kHz, constant force of 29–61 N/m, and radius of curvature of 5 nm to 10 nm. To capture images by AFM, 5 µL of each sample was deposited on the atomically flat surface of a freshly cleaved mica, and excess moisture was removed with an argon flow. The images were obtained in intermittent contact mode (tapping mode). The scan was carried out at a speed of 1 Hz and a resolution of 512 × 512 pixels. The quantitative analysis of the images was accomplished using the “section of analyses” applicative of the system (v.5.30r3.sr3), and a minimum of 10 images of each sample were analyzed to ensure the reproducibility of the results. They were presented as the mean and standard deviation of 50 analyzed particles. Additional sizes and the polydispersion measurements of nanoparticles were assessed and calculated by Quantikov 92-99^®^, a microstructural analyzer for Windows.

### 2.6. Evaluation of Efficiency and Encapsulation Percentages

The encapsulation efficiency and drug loading were determined following the measurement of the total amount of ABZ present in the NCs, the amount of non-encapsulated ABZ (present in the external aqueous phase of the final dispersion), and the total amount of ABZ used to prepare the formulation. The amount of non-encapsulated ABZ was determined after the ultrafiltration–ultracentrifugation of ABZ-loaded NCs using Microcon filters (Amicon^®^, 100,000 Da, Millipore, Burlington, MA, USA). ABZ-loaded NCs were centrifuged at 300× *g* for 10 min (Centrifuge 5415 D, Eppendorf), as represented in Figure 2A. A 100 µL ultrafiltrate aliquot was collected and diluted under vortex agitation in 1 mL of acetonitrile, and the ABZ concentration was determined by UV spectroscopy at 238 nm using the methodology described above. The total amount of ABZ present in the NCs dispersion was determined by diluting 100 µL of NCs in 10 mL of acetonitrile and homogenized under vortex agitation to disrupt the NC structure (MS1, IKA Brazil, Campinas, SP, Brazil). After that, the samples were centrifuged at 300× *g*, and the amount of ABZ present in the supernatant was quantified by UV spectroscopy at 238 nm. All analyses were carried out in triplicate, with three readings collected for each sample. NC formulations were prepared containing 0.5, 1.0, and 5.0 mg/mL of ABZ. The percentage of loading was calculated as the difference between the total amount of ABZ in the final NC dispersion and the amount of non-encapsulated ABZ (present in the ultrafiltrate) divided by the total amount of ABZ quantified in the NC dispersion × 100. The ABZ encapsulation efficiency is the amount encapsulated divided by the ABZ weighted to prepare NCs × 100, considering losses in the process. The payload is the mass ratio of encapsulated ABZ to the NC total mass of excipients.

### 2.7. Determination of ABZ Release In Vitro

The assessment of in vitro free ABZ dissolution and ABZ release profiles from the NCs was performed according to the equilibrium reverse dialysis technique as described by Magalhães et al. (1995) [43]. Briefly, 0.5 mg of an ABZ amorphous powder or 5 mL of NCs was directly diluted in 195 mL of a 0.9% NaCl solution (under sink conditions), where 6 dialysis bags with pores of 12000–14000 Da containing 1 mL of the same 0.9% NaCl solution had previously been immersed. The dialysis bags were kept in equilibrium at 37 °C with the external saline solution for 2 h, before adding the NCs to the external medium. At pre-defined time intervals (0.25, 0.5, 1.0, 2.0, 4.0, and 10.0 h), one of the dialysis bags was removed from the container, and the amount of ABZ present in the bag was determined by UV spectrophotometry at 238 nm, as represented in Figure 2B. The release experiments were carried out in a water bath at 37 °C with constant stirring (Bath Dubnoff mod.144, Fanem, Brazil). The experiment was conducted in triplicate, and each sample was quantified in duplicate.

### 2.8. In Vitro Activity of Albaconazole Nanocapsules (Epimastigote Inhibition Assay)

The Colombian and Y strains were used to determine the in vitro activity of ABZ NCs against epimastigote forms of *T. cruzi*. Parasites were cultured at 28 °C in liver infusion tryptose (LIT) medium supplemented with (10% *v*/*v*) of fetal bovine serum (FBS) in a biochemical oxygen demand incubator. Cultures were started at a cell density of 2 × 10^6^ epimastigotes/mL, with the drug added 72 h later. Cell density was assessed by counting in a Newbauer chamber. Free ABZ was added as a DMSO solution. The DMSO final concentration did not exceed 0.1% (*v*/*v*). As previously determined in a pilot protocol carried out by our group, this DMSO concentration had no effect on epimastigote proliferation. The experiment was conducted in quadruplicate wells and repeated twice (*n* = 8).

### 2.9. Trypanosoma cruzi Strain and Evolutionary Forms

The Y strain is Tc II DTU (Discrete Typing Unity) of *T. cruzi* [44,45]. It was used to infect mice and simulate the acute phase of the murine disease. The Y strain is partially resistant to BZN [46,47]. The Y strain induces high parasitemia in mice, has a fast infection course, and has high animal mortality rates around the 11th to 13th day post-infection. These characteristics make this an excellent model for the in vivo screening of potential drugs with activity against *T. cruzi*. Trypomastigote forms of *T. cruzi* were collected on the day of parasitemia peak from the blood of experimentally infected mice.

### 2.10. Experimental Animals and Ethics

Thirty-day-old female albino *Swiss* mice, weighing around 20 g, from the Central Animal Facility of the Federal University of Ouro Preto, were used. All procedures were conducted in accordance with the guidelines of the National Council of Animal Experimentation Control (CONCEA), following approved protocols CEUA/UFOP n°2015/50, 2012/70/(UFOP/n°2004/08, n°2005/07). During the experiments, the animals were kept in the Central Animal Facility and had free access to food and water ad libitum.

### 2.11. Maximal Tolerated Albaconazole Dose in Healthy Mice

The maximum tolerated dose (MTD) of free ABZ and ABZ-NCs was evaluated using non-infected mice weighing approximately 20 ± 2 g. Surviving animals were monitored for 72 h. Free ABZ solution and NC formulations were administered via the intravenous route. The maximal volume administered to the animals was 0.2 mL, except when larger volumes were required, where the total volume was divided into applications of 0.2 mL at 20-min intervals. To carry out the DMT experiments, groups of 10 mice were treated with different doses of free ABZ (25, 30, 35, and 40 mg/kg) or encapsulated ABZ (80, 120, and 200 mg/kg). A group of five animals was used for treatment with 500 mg/kg of encapsulated ABZ. All the formulations were freshly prepared, and the free ABZ solution and ABZ-NC concentrations were 2.5 mg/mL and 5.0 mg/mL, respectively.

### 2.12. Efficacy Evaluation in the Acute Phase in Infected Mice

In this study, the murine *T. cruzi* experimental model of acute infection with the Y strain was used [48]. This strain is partially sensitive to benznidazole [46,47]. Animals were inoculated intraperitoneally with 10,000 blood trypomastigotes per animal. The blood trypomastigote inoculum came from successive passages in mice. The infected animals used in the in vivo experiments were evaluated daily to determine survival. Parasitemia was assessed daily by fresh blood examination (FBE) until complete parasitemia clearance. Blood was collected from the animal’s tail vein. The number of parasites was counted according to the technique described by Brener [46] under a light microscope by counting 50 fields on a 22 × 22 mm microscope slide. The counted number of parasites was used to build the parasitemia curve. Surviving animals were sacrificed 180 days after infection. For the post-treatment cure criteria, a fresh blood test and blood culture were performed according to Chiari’s technique (1989) [49].

### 2.13. Treatment

After confirming parasitemia (approximately 4 days post-inoculation), the formulations were administered for 20 consecutive days [46]. Each experimental group was composed of 10 animals. Control groups were treated with 5% (*wt*/*v*) glucose solution, blank NCs, free ABZ solution, and blank solution. A positive control group was treated by gavage with oral benznidazole (100 mg/kg/day). Different dosages, routes of administration, and treatment durations were utilized to evaluate the effects on parasitemia. Some groups were treated by gavage with controls and 20 mg/kg of ABZ coarse suspension for 20 consecutive days, and other groups were treated by the subcutaneous (SC) route with increasing doses (20, 40, 80, and 120 mg/kg) for the same duration. Another group was treated with 20 mg/kg of ABZ-loaded NCs for 20 consecutive days by the intramuscular (IM) route. The maximal volume administered was 0.2 mL. When a volume larger than 0.2 mL was required, the total volume was divided into applications of 0.2 mL administered at 20-min intervals.

### 2.14. Statistical Analysis

The hydrodynamic diameter, zeta potential, ABZ efficiency of encapsulation of NCs, and release data points were compared using an unpaired Student’s *t*-test using the EpiInfo analysis program version 6.04. Comparisons between the pre-patent and patency periods, peak parasitemia (during and after treatment), and average survival times were performed using Student’s *t*-test. To compare the parasitemia curves, the non-parametric Kolmogorov and Smirnov test was used, which compares the area under the parasitemia curve between two samples. Comparisons were made between the curves obtained from untreated and treated mice, as well as between the curves obtained from mice treated with the different formulations. One-way ANOVA with Dunn’s multiple-comparisons post-test (95% confidence level) was used for in vitro activity data using Prisma^®^ 8.0 software. The differences were considered significant with *p* < 0.05 and a 95% confidence level.

## 3. Results

### 3.1. Nanocapsule Characterization

The NC size measurement was performed by two techniques: DLS in a liquid medium and AFM on dried samples dispersed on mica plates. The pH of NC dispersion and the zeta potential of NC surface containing different concentrations of ABZ were also evaluated, and the results are shown in Table 1. The analysis was performed using freshly prepared formulations.

The hydrodynamic diameter of ABZ-NCs increased significantly (*p* < 0.05) with increased ABZ concentrations; however, after loading 5 mg/mL, the maximal payload seemed to be surpassed. After some days, the ABZ crystals grew in the dispersion. All the formulations containing 0 to 1 mg/mL of ABZ were monodisperse, considering a polydispersity index lower than 0.3 during the first week after preparation, and only 5 mg/mL showed instabilities after this storage time. The NC sizes increased until reaching 1 mg/mL, compared to blank NCs. At 5 mg/mL, there is a reduction in mean sizes, probably due to the unloaded ABZ macroscopic crystal that precipitates out of the nanostructure. As a result, 5.0 mg/mL NCs of ABZ show a reduced size, and the PDI is closer to the value of blank-NCs, since the macroscopic crystals of ABZ are not detected by equipment that has a maximum particle detection limit of 3 μm. The pH values of liquid dispersion vary with increasing ABZ concentrations due to its alkaline character. The zeta potential of NCs is negative without significant changes (*p* > 0.05), ranging from −49.2 to −59.5 mV, indicating a low association of ABZ with the surface of NCs. Thus, ABZ is probably associated with the oily nucleus of the NCs, interfering little in the zeta potential. It seems that the optimal loading concentration of ABZ is between 1 and 5 mg/mL.

**Figure 3:**
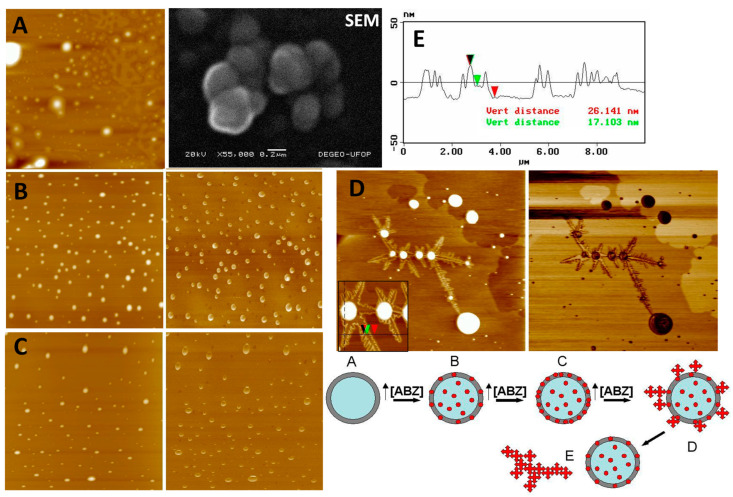
Scanning electron microscopy image (SEM) of ABZ-NCs (1 mg/mL) (in black). Atomic force microscopy (AFM) images showing height (on the left) and phase (on the right), showing (**A**) blank NCs (scan size, 40 µm × 40 µm); (**B**) ABZ-NCs, 0.5 mg/mL (scan size, 40 µm × 40 µm); (**C**) ABZ-NCs, 5.0 mg/mL (scan size, 40 µm × 40 µm); (**D**) ABZ-NCs, 5.0 mg/mL stored for a few hours, showing spherical structures spread on mouse plates surrounded by crystals (scan size, 40 µm × 40 µm); crystal growth, with image insert (scan size, 10 µm × 10 µm) of one crystal size measurement; and (**E**) measurement of ABZ crystals formed on mica plates using AFM equipment software (v.5.30r3.sr3). In the bottom figure, a schematic representation of the ABZ crystallization hypothesis growing from the NC nucleus is shown, indicating the morphological changes following increased ABZ concentration in NC formulations until supersaturation is reached with the presence of nanometric ABZ crystals.

Scanning electron microscopy (SEM) was used to observe the morphology of NCs containing 1 mg ABZ/mL (Figure 3). Spherical nanostructures are clearly shown. However, this method was not demonstrated to be suitable since the coating of the nanostructures with gold was not uniformly distributed, causing deformation in the structures and even the partial melting of the sample under the electron beam. The NCs can be observed to be slightly deformed, although a three-dimensional spherical appearance is evident. Thus, the analysis of the morphological features of NCs was performed by AFM, since this technique requires no covering of samples (Figure 3A–E). AFM analysis corroborated the physicochemical analysis in the liquid media, indicating that, probably, concentrations of ABZ above 1.0 mg/mL are adsorbed on the NC surface, reaching a saturation level from which the drug precipitates in the external aqueous media in the form of ABZ crystals. In Figure 3, showing height and phase images collected from NC samples containing 0–5 mg of ABZ/mL, nanometric crystals can be observed around NCs (Figure 3D,E). These images show ABZ crystallization from NC nuclei. The ABZ solution allowed to evaporate deposited on mica produced the same standard of microcrystal structures. The crystals and amorphous structures observed in the ABZ-loaded NCs were not observed in blank NCs. The supersaturation of ABZ in the dehydrated samples upon AFM analysis probably induced the rapid crystallization of ABZ, as also observed in NC samples of 5.0 mg/mL in a liquid medium. The schematic representation in Figure 3 is based on the AFM results. In Table 2, the mean geometric diameter measured using AFM images (topographic profiles, Figure 3E) for NC samples was larger than the diameters in the liquid media. This hypothesis is in agreement with observations of NC flattening on the mica surface reported previously [50].

The results presented in Table 2 show high encapsulation efficiency for this process, with the highest percentage for 1 mg/mL. The drug loading, the difference in the final NC dispersion between the drug loaded and unloaded, is also very high. These results indicate that ABZ has more affinity for the oily core of NCs than for the external aqueous medium and that it therefore associates better with NCs due to their high lipophilicity. The estimated *c*log *P* is 2.564 (computed by XLog*P* 3 3.0, PubChem release 14 October 2021). The encapsulation efficiency of ABZ at 5 mg/mL is significantly reduced (*p* < 0.05) in relation to other formulations. Thus, values greater than 1 mg/mL of ABZ probably saturate the system, resulting in the precipitation of ABZ in the external medium, which is responsible for low encapsulation efficiency values at 5 mg/mL concentration (Figure 3D). This result is in agreement with the AFM analysis (Figure 3, SEM image).

### 3.2. Albaconazole Release Profiles

**Figure 4:**
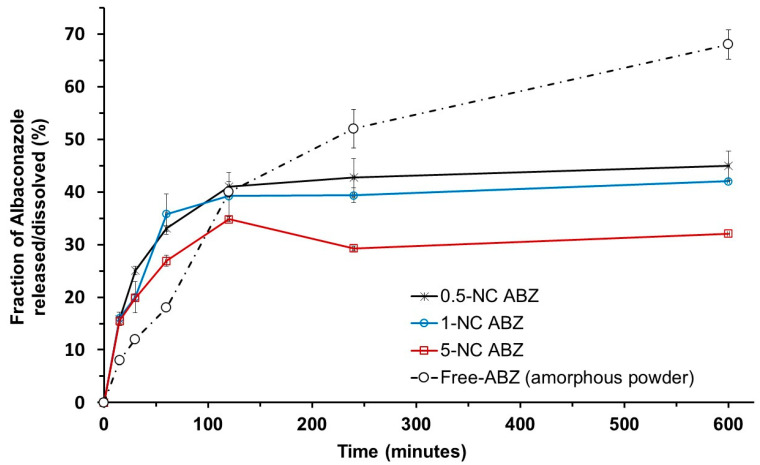
Dissolution profile and release profiles of ABZ from polymeric PCL nanocapsules for three ABZ concentrations: 0.5, 1, and 5 mg/mL. The experiment was performed in saline at 37 °C under sink conditions (20% saturation solubility) by the reverse dialysis method. Each point represents mean values and standard deviations.

Figure 4 shows the results for the release profile of ABZ from NCs. The method used to determine the release profile (reverse dialysis) in vitro of encapsulated ABZ was efficiently used in this experiment (Figure 2C).

The release of ABZ from NCs to the external medium was relatively fast, incomplete, and biphasic. From 0 to 1 h, the release profile of the 0.5, 1.0, and 5.0 mg/mL ABZ formulations followed the same pattern, with a “*burst*” release effect for all the NCs (approximately 15%). After 120 min, the formulations containing 0.5 and 1 mg/mL of ABZ showed similar release rates with faster release compared with 5 mg/mL of ABZ. Around 40% of ABZ was released into the external media in less than 4 h. It is observed that after 2 h, a slower profile phase started and continued until it reached a plateau. Up to 10 h, the release of ABZ was incomplete in this media. The reprecipitation of ABZ appeared to occur in the release medium with 5 mg/mL NC formulation after 120 min, which reduced the concentration of ABZ in the release medium. This is also in accordance with the results of encapsulation efficiency (Table 2) and AFM analysis (Figure 3). Free ABZ dissolution was slowed in the first hour in this medium. However, the dissolution continued until it reached 66% in 10 h. NCs were able to retain ABZ even under sink conditions.

### 3.3. Albaconazole Activity In Vitro Against T. cruzi Epimastigotes

**Figure 5:**
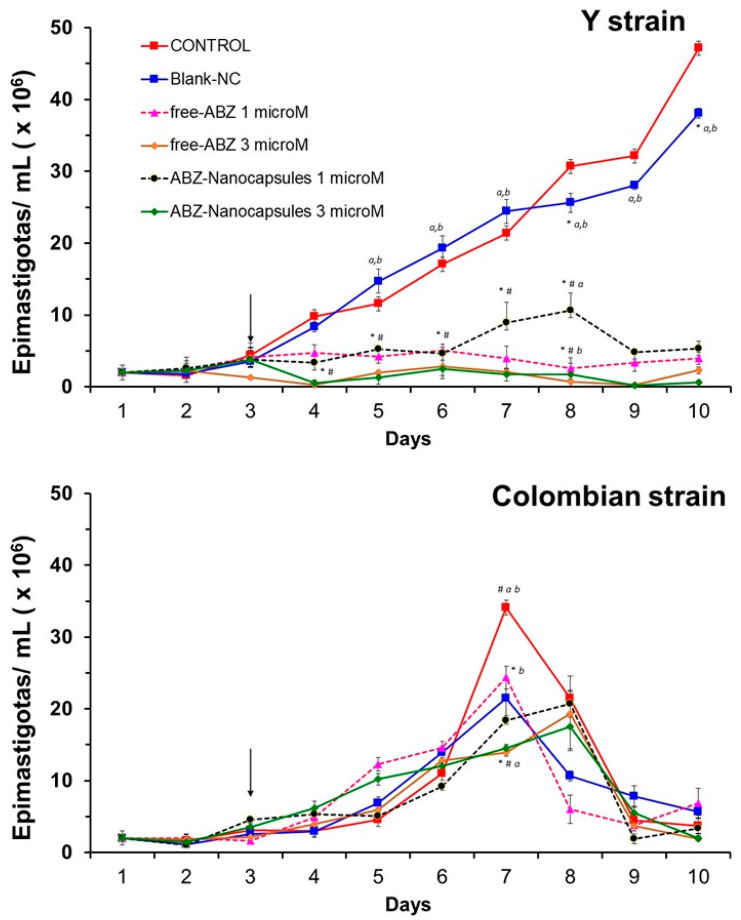
Effects of free ABZ and ABZ-NC in two concentrations (1 and 3 µM) of ABZ on the proliferation of epimastigote forms of the Y and Colombian strains of *T. cruzi*. The arrow indicates the day that formulations were added to the culture media. The experiment was conducted in quadruplicate wells and repeated twice (*n* = 8). The statistical significance (*p* < 0.05) is represented by symbols inside the graphs: * different from control; # different from blank NCs; *a*, different from free -ABZ; *b*, different from ABZ-NCs.

The effects of ABZ against epimastigote forms of *T. cruzi*, Y and Colombian strains, are shown in Figure 5. An inhibitory action of ABZ was observed against the Y strain (*p* < 0.05), compared to untreated control cultures and cultures treated with blank NCs. This inhibitory action began 24 h after the addition of ABZ and was not dose-dependent, as no marked differences were observed between 1 and 3 μM. No differences were observed in the activity of free or encapsulated ABZ regarding culture growth. Free ABZ and ABZ-NCs were ineffective in controlling cell growth when added to cultures of the Colombian strain, even when higher doses were tested (3 µM), confirming the resistance of this strain [46,51].

### 3.4. General Toxicity Evaluation by Intravenous Route

According to the graph of the percentage of survival versus the concentration of ABZ administered via the IV route represented in Figure S3 (Supplementary Materials), the LD_50_ of free ABZ is 30.4 mg/kg. It was not possible to experimentally obtain the LD_50_ value for ABZ encapsulated in NCs, as even at high concentrations of the drug, no deaths were observed within 72 h (LD_50_ > 500 mg/kg). Therefore, encapsulation proved to be a very useful alternative for reducing the general toxicity of ABZ, since DL_50_ was increased more than 15-fold, as can be seen in Table 3. It shows the adverse effects observed in animals after the intravenous administration of different doses of ABZ. It can be observed that with the encapsulation of ABZ, the intensity and number of side effects are significantly reduced (*p* < 0.05). Free ABZ at 40 mg/kg induced death in all animals, and the double dose (80 mg/kg) encapsulated induced no clinically observed adverse effects in 72 h. Only 500 mg/kg of ABZ-NCs induced a significant increase in serious adverse effects, but no death was observed. Thus, nanoencapsulation dramatically reduces the toxicity of ABZ administered intravenously.

### 3.5. In Vivo Efficacy in Infected Mice

The efficacy of ABZ in the mouse model infected with the Y strain partially resistant to BNZ was evaluated following different parameters: routes of administration, doses, and dosage regimen. The main results are shown in Table 4.

Blank-NCs and parenteral suspension excipients had no significant effect on infected animals’ parasitemia levels (*p* > 0.05) compared to the untreated control that received only isotonic glucose. Benznidazole classical treatment for mice induced 100% survival and negative parasitological tests for 120 days. Free ABZ suspensions were administered via two different routes (PO and SC) with a treatment regimen of 20 uninterrupted days, in addition to being tested via SC at three different doses (20, 80, and 120 mg/kg). Free ABZ administered by the oral route shows no advantageous reduction in parasitemia compared with subcutaneous at the same doses. According to Table 4, the efficacy was significantly higher via SC than oral administration at a dose of 20 mg/kg for the patent period (*p* < 0.05) regarding parasitemia during (*p* < 0.05) and after (*p* < 0.05) the treatment. Survival was 100% for both routes of administration. However, using the SC route, 70% of the animals continued to have negative parasitemia up to 20 days after treatment, while 100% showed patent parasitemia and relapses after oral treatment. At a dose of 80 mg/kg SC, there was a significant reduction in the patent period (*p* < 0.02), without the reactivation of parasitemia after treatment in 100% of the animals. However, survival was 30% lower compared to the 20 mg/kg dose, demonstrating the toxicity of ABZ during long-period SC administration, as the animals died in the second week after starting treatment with negative parasitemia values. There was no significant difference in maximum parasitemia values for surviving animals at the three doses used (20, 80, and 120 mg/kg) via SC (*p* > 0.05) (Table 4). At a dose of 120 mg/kg of free ABZ, there was a significant reduction in the mean survival time (*p* < 0.05), with the animals all dying during the first week of treatment with negative parasitemia. This result indicates that this SC dose of free ABZ was highly toxic to mice, in agreement with previous experiments on the maximal tolerated dose by intravenous administration. The maximum parasitemia, however, was at the same level as the 80 mg/kg dose (Table 4).

Due to the failure of ABZ-NC administration by IM to induce a significant reduction in parasitemia during the patent period, the studies with higher doses of ABZ continued only via the SC route for NCs. The encapsulated ABZ at a dose of 40 mg/kg divided into two daily doses of 20 mg/kg resulted in a longer time for parasitemia reactivation after the end of treatment compared with a single daily dose of 40 mg/kg. However, survival was reduced. At doses of 80 and 120 mg/kg, an increase in efficacy was observed with a significant reduction in the patent period (*p* < 0.05) and the elimination of blood parasites for a period of more than 120 days in 20% and 60% of treated animals, respectively (Table 4). No toxic effects were observed during and after treatment with ABZ-NCs (40–120 mg/kg). This significantly differentiated the formulations, because free ABZ at the same doses induced the death of 30% and 100% of the animals, respectively. The rapid and effective elimination of parasitemia with doses of 80 and 120 mg/kg ABZ-NCs was evident (Figure S4, Supplementary Materials). At the dose of 120 mg/kg ABZ-NCs, there was no reactivation of parasitemia up to 60 days after the end of treatment (Table 4).

## 4. Discussion

The therapeutic management of Chagas disease requires new drugs, as the standard-of-care medicines available cause serious adverse effects and have limited efficacy. Benznidazole has low efficacy in the chronic phase of the disease, and only this drug is available in many endemic countries of Latin America [2,4]. In the search for new options, in this study, we developed a safe formulation of ABZ loaded into biodegradable polymeric biodegradable nanocapsules (NCs) (Figure 1), which can be administered by oral and parenteral routes (IV, SC, IM). To date, only tablets and conventional gelatin capsule dosage forms have been reported [18], and these formulations have been used only in clinical trials and are not commercially available. Considering the solubility limitations of ABZ (0.001 mg/mL at 30 °C pH 1.2) [52] and its high lipophilicity, we successfully developed a new formulation of ABZ that could be administered parenterally by IM, SC, IV, and even the oral route, composed of biodegradable and biocompatible excipients.

As reported previously, the efficacy of ABZ in the murine model is reduced compared to the efficacy in the dog model [23]. Similarly, herein, parasitological and serological cure in mice treated with ABZ were not obtained in 100% of cases, as previously reported in dogs [23]. Guedes et al. (2004) demonstrated that only a regimen of multiple daily doses was able to improve the therapeutic efficacy in dogs, indicating that ABZ also possesses unfavorable pharmacokinetic properties in this animal model [23].

ABZ appears to have been abandoned in the repurposing strategy against Chagas disease, likely due to its short half-life in animals, in favor of another azole derivative, E-1224, a pro-drug of ravuconazole. Recently, proof-of-concept clinical trials with E-1224 indicated no efficacy [53]. As previously discussed, the rapid suppression of parasitemia and low curative rates of azole derivatives are probably associated with unfavorable pharmacokinetic characteristics in mice that reduce the amount of the drug available in tissues for the eradication of intracellular parasites [11]. For ABZ, a long terminal half-life has been reported in dogs (51 h) and monkeys (24 h) [54]. However, pharmacokinetic studies on ABZ in humans have suggested a prolonged half-life [17,18].

ABZ is a potent lipophilic ergosterol inhibitor that has been studied clinically as an antifungal agent. The inhibition of parasite sterol C14α-demethylase has also been identified as the mechanism of action against *T. cruzi*. Urbina et al. reported potent in vitro activity of ABZ against epimastigotes of the Y strain of *T. cruzi* (MIC 30 nM). In addition, intracellular amastigotes cultured in Vero cells were even more susceptible (IC_99_ of 10 nM and IC_50_ of 1 nM) [22]. Furthermore, no effect was seen on the viability and proliferation of the Vero host cells up to the highest concentration tested (100 nM), indicating a specific antiparasitic activity, as well as a suitable selectivity index (>100). In another study, ABZ induced no reduction in the viability of rat primary hepatocytes at doses up to 40 µM [52]. The PCL NCs developed in this study have been shown to possess low cytotoxicity toward host cells. Their CC_50_ is higher than 248 µg/mL, > 1000 µg/mL, and >1000 µg/mL against the Vero, HepG2, and Caco2 cell lines, respectively, in vitro [55].

More recently, the cardiac toxic effects of supratherapeutic ABZ exposure with multiple and sustained high doses on the ECG parameters of 24 healthy subjects were investigated in clinical trials [17]. Pharmacokinetics studies on humans have indicated that ABZ is rapidly absorbed (T_max_ 5–22.5 h) after an oral dose of 400 mg every 8 h, reaching a mean C_max_ of 11.9 ng/mL after 5 days, and its metabolite, 6-hydroxialbaconazole, reaches a C_max_ of 636 ng/mL. This study concluded that this dosing schedule was well tolerated and the adverse effects were mild, without safety issues [17]. Another clinical study in healthy volunteers demonstrated that capsules had higher bioavailability than tablets, indicating that these two dosage forms were not bioequivalent. This study points out the importance of developing suitable dosage forms for ABZ [18].

In mice, we demonstrated herein that the dosage form can dramatically change the tolerability of ABZ. Nanoencapsulation improves ABZ safety and probably its biopharmaceutical properties, providing prolonged release following the investigated administration routes. The NC formulation is stable and biodegradable, produces reproducible and monodispersed particles, and has suitable sizes and prolonged-release properties. NCs possess an excellent ability to entrap ABZ at a high encapsulation efficiency to be administered by the different parenteral routes tested in this study, as well as by the oral route. In terms of in vitro activity, ABZ exerts suppressive effects against epimastigotes of the Y strain, which is in agreement with previously reported data. However, no significant in vitro effect was observed against the BZN-resistant Colombian strain. This finding is similar to the data obtained by Guedes et al. (2004) against the Berenice-78 strain in vivo, a BZN-sensitive strain [23]. Thus, natural *T. cruzi* resistance to triazole exists, as has been documented previously [56]. No difference was observed between free ABZ and ABZ-NCs against epimastigotes, which is to be expected, as in vitro acellular conditions do not adequately simulate the complexity of in vivo absorption, distribution, metabolization, and elimination. In vitro, the poorly soluble drugs, such as ABZ loaded into nanocapsules, have an incomplete and slow-release rate, as demonstrated here (Figure 4). After SC administration in vivo, the NCs may form a depot of ABZ. In fact, nanocarriers are known to alter drug absorption and distribution steps in vivo. Thus, animal models are more suitable for evaluating the efficacy and toxicity effects of drug encapsulation.

The most recent clinical trials with CYP51 inhibitors indicate that they initially eliminate parasites from blood, but they are not able to maintain this effect after the end of treatment [4,53]. These clinical trials evidence the same effect that we observed in mice with ABZ in this study. As hypothesized by Dumoulin et al. (2020), the anti-*T. cruzi* activity of azoles induces no sterile cure, which opens up the possibility of distinct populations of parasites and/or a heterogeneous environment within a single host underlying treatment failure [57]. The resistance of *T. cruzi* to the azole class of drugs has not yet been reported and merits further investigation.

Effects were markedly different concerning tolerability and reductions in parasitemia relapses in vivo, particularly at doses of 80 and 120 mg/kg, with low levels of parasitemia and a long time to parasitemia reactivation seen with ABZ-NC treatment compared to free ABZ. Protocols for once- and twice-day doses were similarly effective, demonstrating that NCs prolonged ABZ residence time in the body. Therefore, a dose-dependent therapeutic response was observed when ABZ-NCs were administered subcutaneously. These data indicate that the pharmacokinetic profile of the drug was probably altered with the use of NCs. Furthermore, the doses of ABZ required to eliminate parasitemia were significantly higher for NCs than for free ABZ. This may be due to the formation of an NC deposit in the subcutaneous tissue that did not immediately make the concentration of ABZ available to eradicate parasites. It could be suggested, however, that NCs can sustain blood levels of ABZ better than free ABZ, allowing a slower relapse of parasitemia. This hypothesis, which should be confirmed further by biodistribution studies, considers that the NCs can retain ABZ in the injection site, releasing fractions of ABZ, and reducing adverse effects. The in vitro release profile of ABZ from NCs showed that a maximum of 45% of the drug was released in the first 10 h, a result that corroborates our hypothesis. The differences between free ABZ and ABZ-NCs can be attributed to changes in the biodistribution of the drug when administered by the SC route in free or encapsulated form, which merits further investigation.

No parasite elimination was achieved with any formulation in our experimental conditions, indicating a need to carry out future studies with higher doses, as well as experiments in combination with other drugs. ABZ has suppressive effects on *T. cruzi*, and encapsulation prolongs this suppression. From a pharmacological point of view, NCs significantly improved the safety of ABZ in vivo. Hence, appropriate formulations must be developed to control the release of ABZ and optimize the dosage regimen, which may be helpful in the extended treatment regimen for Chagas disease and improve adherence to treatment in patients in the chronic phase.

The most promising effect observed in this work was related to the overall reduced toxicity of ABZ in the encapsulated form. Although a parasitological cure was not obtained in the animals treated with the new ABZ-NC formulations, the results were positive concerning subcutaneous administration, which allowed more sustained action. This effect of NCs promoting prolonged release and reduced toxicity has been observed in different studies [58,59,60], which attribute the toxicity reduction to the reduced access of NCs to vital organs, such as the heart, reducing, for example, the cardiotoxicity [30,58]. Our results in the present study corroborate this hypothesis, even using another administration route, i.e., SC. Therefore, further in-depth studies on tissue infection in animals treated with different doses and formulations of encapsulated ABZ are necessary.

As Tarlenton (2001) argues, parasite elimination from tissues is one of the treatment goals in the chronic phase of Chagas disease and allows a significant reduction in symptoms and mortality in humans [61]. The present study’s data reinforce the importance of developing new dosage forms for this class of drugs to improve biopharmaceutical characteristics.

## 5. Conclusions

In this study, we developed innovative formulations of ABZ, an investigational triazole, in biodegradable polymeric nanocapsules. Originally, these NCs were able to be delivered by parenteral routes, as well as orally. The use of SC injections emerged as an interesting alternative to administer anti-*T. cruzi* drugs and obtain a depot in this tissue. The ABZ-NCs are stable and monodisperse in size within the nanometrical range. NCs have an adequate ability to load ABZ with high encapsulation efficiency. It was evidenced that NCs dramatically improve in vivo ABZ safety by the intravenous route and SC routes, even at high doses. ABZ-NCs improved mice survival compared to free ABZ, induced the fast elimination of parasitemia, and prolonged the anti-*T. cruzi* effect after the end of treatment in a murine model. This indicates that NCs represent a promising strategy to circumvent the biopharmaceutical limitations of ABZ. The encapsulation of ABZ provides a dosage form that is adaptable to various administration routes. This preliminary pre-clinical study in mice can pave the way for deeper investigations into its effects on parasite burden in different organs, potential immunological modulation, and further toxicological investigations.

## Figures and Tables

**Table 1:** Physicochemical properties of albaconazole nanocapsules.

Formulation	ABZ mg/mL	Hydrodynamic Diameter ± SD ^2^ (nm)	PDI ^3^	AFM ^4^ Mean Size ± DP ^1^ (nm)	Zeta Potential ± SD (mV) *	pH ± SD ^2^
Blank NCs	0	171.4 ± 0.8	0.127 ± 0.022	336 ± 144	−50.1 ± 1.2	6.69 ± 0.01
0.5 NCs	0.5 *	201.5 ± 0.4	0.096 ± 0.019	195 ± 54	−49.2 ± 3.4	6.44 ± 0.08
1 NCs	1.0 *	225.9 ± 2.1	0.193 ± 0.022	-	−59.5 ± 5.1	7.02 ± 0.04
5 NCs	5.0 ^5^	155.9 ± 0.3	0.130 ± 0.004	250 ± 57	−50.5 ± 3.0	7.10 ± 0.20

^1^ EE, encapsulation efficiency; ^2^ SD, standard deviation; ^3^ PDI, Polydispersion Index, ^4^ AFM, atomic force microscopy; ^5^ macroscopic crystallization of ABZ after 7 days post-preparation. Statistical analysis was performed using Student’s *t*-test, comparing ABZ-NCs with blank NCs (* *p* < 0.05).

**Table 2:** Albaconazole association with poly-ε-caprolactone nanocapsules.

ABZ Formulation	ABZ mg/mL (Feed)	ABZ in NCs (mg/mL ± SD ^2^) (Real)	EE ^1^	% Drug Loading *	Payload ^#^ (µg/mg)
0.5 NCs	0.5	0.36 ± 0.18	71.4 ± 4.1	99.79± 0.38	7.83
1 NCs	1.0	1.17± 0.03	117 ± 3.6	94.27± 0.15	25.43
5 NCs	5.0	1.39 ± 0.32	27.9± 1.9 *	94.18± 0.32	30.22 *

^1^ EE, encapsulation efficiency; ^2^ SD, standard deviation; * in freshly prepared NC formulations; **^#^** calculations and definitions are provided in the methodology.

**Table 3:** Adverse effects caused by intravenous administration of free ABZ or ABZ-loaded nanocapsules.

		Evaluated Parameters			
	Dose	Ataxia	Respiratory Changes	Convulsion	Survival *
Free ABZ (intravenous solution) ^1^	25 mg/kg	-	-	-	10/10
	30 mg/kg	+++	+++	++	5/8
	35 mg/kg	-	-	++	2/10
	40 mg/kg	-	-	+++	0/9
ABZ NCs ^2^	80 mg/kg	-	-	-	10/10
	120 mg/kg	++	+	-	10/10
	200 mg/kg	++	+	-	8/8
	500 mg/kg	+++	+++	-	5/5

^1^ ABZ solution (2.5 mg/mL); ^2^ freshly prepared ABZ nanocapsules (5 mg/mL); * evaluated for 72 h. The symbols represent (+++) strong, (++) moderate, and (+) mild effects.

**Table 4:** Summary of the effects of treatment with free ABZ and ABZ-loaded nanocapsules on infected mice (Y strain of *T. cruzi*).

Dose (mg/kg/Day)		Via	Survival	MST ^1^ (Days)	Patent Period (Days ± SD ^2^)	Maximal Parasitemia Level (×1000) ± SD ^2^		Negative Parasitological Tests ^3^	
						During ttm.	After ttm.	d 90	d 120
Control (untreated)		SC	0/10	11.4 ± 1.3	8.3 ± 1.5	1418 ± 969	-	-	-
Blank-NCs		SC	0/10	10.2 ± 7.6	7.2 ± 7.6	1427 ± 3082	-	-	-
DMA/PEG 300 solution		SC	0/10	8.4 ± 4.9	5.4 ± 4.9	1976 ± 420	-	-	-
Benznidazole (100 mg/kg/day)		PO	10/10	>60	0	0.5 ± 0 *	nd	10/10	10/10
Free ABZ	20	PO	10/10	>60 *	30 ± 4.8 ^#^	27 ± 29 ^#^ *	7.5 ± 4.3	-	-
	20	SC	10/10	>60 *	9.8 ± 12.4 ^#^	0.5 ± 1.6 *	0.5 ± 1.7	-	-
	80	SC	7/10	>60 ^4^ *	1.4 ± 0.8 ^#^	0 *	0	3/7 ^#^	0/7 ^#^
	120	SC	0/10	4.7 ± 1.6 ^#^	1 ± 0.0	0 *	nd	-	-
ABZ-NCs	20	IM	9/10	>60 ^5^ *	27.1 ± 3.1 ^#^	318.5 ± 558.5 ^#^	9.0 ± 11.5	-	-
	20	SC	8/10	>60 ^6^ *	30.1 ± 6.1 ^#^	103.0 ± 104.5 ^#^	12.7 ± 16.9 **	-	-
	40 (2 × 20)	SC	9/10	>60 ^7^ *	16.5 ± 14.6 ^#^	3.3 ± 5.6 *	3.9 ± 8.6	-	-
	40	SC	10/10	>60 *	22.6 ± 11.9 ^#^	0.5 ± 1.6 *	2.5 ± 3.5	-	-
	80	SC	10/10	>60 *	22.4 ± 12.8 ^#^	4.0 ± 12.6 ^#^ *	2.0 ± 2.6	2/10 ^#^	2/10 ^#^
	120	SC	10/10	>60 **	1.1 ± 0.3	0 *	0 **	6/10 ^#^ **	6/10 ^#^ **

^1^ MST, mean survival time; ^2^ SD, standard deviation; ^3^ in days after blood culture was performed; ^4^ animals died on 9th, 10th, or 13th day after inoculation; ^5^ animal died on 33rd day after inoculation; ^6^ animals died on 21st and 27th day after inoculation; ^7^ animal died on 6th day after inoculation; * significantly different from the untreated group (*p* < 0.05); ** significantly different from free ABZ group (*p* < 0.05); ^#^ significantly different from BZN group (*p* < 0.05); nd, not determined. PO, per os (oral); SC, subcutaneous; IM, intramuscular.

## Data Availability

Data are available from the authors.

## Supplementary Materials

The following supporting information can be downloaded at: https://www.mdpi.com/article/10.3390/pathogens14040319/s1, Figure S1: Ultraviolet spectrum showing the maximum absorption peak of ABZ in acetonitrile (5 mg/mL) at 238 nm; Figure S2: ABZ calibration curve in acetonitrile at a wavelength of 238 nm; R2 = coefficient of determination; Table S1: ABZ average absorbance values as a function of its concentration; Table S2: Results of the calibration curve made with pure ABZ, ABZ + NC at 1% and ABZ + NC at 10%; Table S3: Results of the repeatability test to validate the spectrophotometric assay of ABZ at 238 nm; Table S4: Results of the intermediate precision test, in two days, to validate the spectrophotometric measurement of ABZ at 238 nm; Table S5: Accuracy of the ABZ determination method; Figure S3: Survival after treatment with different doses of Albaconazole coarse suspensions and ABZ-NCs; Figure S4: Parasitemia curves after treatment with different doses of Albaconazole loaded in NCs.

## References

[1] PAHO/WHO—Pan American Health Organization (2022). Chagas Disease in the Americas for Public Health Workers-PAHO/WHO. https://www.who.int/campaigns/world-chagas-disease-day/2022.

[2] PAHO/WHO (2019). PAHO/WHO: Guidelines for the Diagnosis and Treatment of Chagas Disease.

[3] Brindley P.J., Hotez P.J., Kamhawi S. (2025). Revisiting What Constitutes a Neglected Tropical Disease?. PLoS Negl. Trop. Dis..

[4] Torchelsen F.K.V.D.S., Mazzeti A.L., Mosqueira V.C.F. (2024). Drugs in Preclinical and Early Clinical Development for the Treatment of Chagas’s Disease: The Current Status. Expert Opin. Investig. Drugs.

[5] Cançado J.R. (2002). Long term evaluation of etiological treatment of chagas disease with benznidazole. Rev. Inst. Med. Trop. São Paulo.

[6] Andrade A.L.S.S., Martelli C.M.T., Oliveira R.M., Silva S.A., Aires A.I.S., Soussumi L.M.T., Covas D.T., Silva L.S., Andrade J.G., Travassos L.R. (2004). Short report: Benznidazole efficacy among Trypanosoma cruzi-infected adolescents after a six-year follow-up. Am. J. Trop. Med. Hyg..

[7] Sosa Estani S., Segura E.L. (1999). Treatment of Trypanosoma cruzi infection in the undetermined phase. Experience and current guidelines of treatment in Argentina. Mem. Inst. Oswaldo Cruz.

[8] Yun O., Lima M.A., Ellman T., Chambi W., Castillo S., Flevaud L., Roddy P., Parreno F., Albajar V.P., Palma P.P. (2009). Feasibility, drug safety, and effectiveness of etiological treatment programs for chagas disease in honduras, guatemala, and bolivia: 10-year experience of medecins sans frontieres. PLoS Negl. Trop. Dis..

[9] Viotti R., Vigliano C., Armenti H., Segura E. (1994). Treatment of chronic Chagas disease with benznidazole: Clinical and serological evolution of patients with long-term follow-up. Am. Heart J..

[10] Duschak V.G. (2016). Targets and patented drugs for chemotherapy of Chagas disease in the last 15 years-period. Recent Pat. Anti-Infect. Drug Discov..

[11] Buckner F.S., Urbina J.A. (2012). Recent developments in sterol 14-demethylase inhibitors for Chagas disease. Int. J. Parasitol. Drugs Drug Resist..

[12] Bartrolí J., Turmo E., Algueró M., Boncompte E., Vericat M.L., Conte L., Ramis J., Merlos M., García-Rafanell J., Forn J. (1998). New azole antifungals. 3. Synthesis and antifungal activity of 3-substituted-4(3H)-quinazolinones. J. Med. Chem..

[13] Sorbera L.A., Bartroli J. (2003). Castañer. Drugs Future.

[14] Seyedmousavi S., Rafati H., Ilkit M., Tolooe A., Hedayati M.T., Verweij P., Lion T. (2017). Systemic Antifungal Agents: Current Status and Projected Future Developments. Human Fungal Pathogen Identification.

[15] Bartroli J., Merlos M., Sisniega H. (2011). Overview of albaconazole. Eur. Infect. Dis..

[16] Guillon R., Pagniez F., Picot C., Hédou D., Tonnerre A., Chosson E., Duflos M., Besson T., Logé C., Le Pape P. (2013). Discovery of a Novel Broad-Spectrum Antifungal Agent Derived from Albaconazole. ACS Med. Chem. Lett..

[17] Dietz A.J., Barnard J.C., van Rossem K. (2014). A randomized, double-blind, multiple-dose, placebo-controlled, dose escalation study with a 3-cohort parallel group design to investigate the tolerability and pharmacokinetics of albaconazole in healthy subjects. Clin. Pharmacol. Drug Dev..

[18] van Rossem K., Lowe J.A. (2013). A Phase I, randomized, open-label crossover study to evaluate the safety and pharmacokinetics of 400 mg albaconazole administered to healthy participants as a tablet formulation versus a capsule formulation. Clin. Pharmacol. Adv. Appl..

[19] Sigurgeirsson B., van Rossem K., Malahias S., Raterink K. (2013). A phase II, randomized, double-blind, placebo-controlled, parallel group, dose-ranging study to investigate the efficacy and safety of 4 dose regimens of oral albaconazole in patients with distal subungual onychomycosis. J. Am. Acad. Dermatol..

[20] Miller J.L., Schell W.A., Wills E.A., Toffaletti D.L., Boyce M., Benjamin D.K., Bartroli J., Perfect J.R. (2004). In vitro and in vivo efficacies of the new triazole albaconazole against *Cryptococcus neoformans*. Antimicrob. Agents Chemother..

[21] Capilla J., Ortoneda M., Pastor F.J., Guarro J. (2001). In vitro antifungal activities of the new triazole UR-9825 against clinically important filamentous fungi. Antimicrob. Agents Chemother..

[22] Urbina J.A., Lira R., Visbal G., Bartroli J. (2000). In vitro antiproliferative effects and mechanism of action of the new triazole derivative UR-9825 against the protozoan parasite *Trypanosoma* (Schizotrypanum) *cruzi*. Antimicrob. Agents Chemother..

[23] Guedes P.M., Urbina J.A., Lana M., Afonso L.C., Veloso V.M., Tafuri W.L., Machado-Coelho G.L., Chiari E., Bahia M.T. (2004). Activity of the new triazole derivative albaconazole against *Trypanosoma (Schizotrypanum) cruzi* in dog hosts. Antimicrob. Agents Chemother..

[24] Girmenia C. (2009). New generation azole antifungals in clinical investigation. Exp. Opin. Investig. Drugs.

[25] Aperis G., Mylonakis G. (2006). Newer triazole antifungal agents: Pharmacology, spectrum, clinical efficacy and limitations. Expert Opin. Investig. Drugs.

[26] Pasqualotto A.C., Thiele K.O., Goldani L.Z. (2010). Novel triazole antifungal drugs: Focus on isavuconazole, ravuconazole and albaconazole. Curr. Opin. Investig. Drugs.

[27] Fávero M.L.D., Bonetti A.F., Domingos E.L., Tonin F.S., Pontarolo R. (2022). Oral antifungal therapies for toenail onychomycosis: A systematic review with network meta-analysis toenail mycosis: Network meta-analysis. J. Dermatol. Treat..

[28] Bartroli X., Uriach J. A clinical multicenter study comparing efficacy and tolerability between five single oral doses of albaconazole and fluconazole 150 mg single dose in acute vulvovaginal candidiasis. Proceedings of the 45th Interscience Conference on Antimicrobial Agents and Chemotherapy: Abs M-722.

[29] Girmenia C., Finolezzi E. (2011). New-generation triazole antifungal drugs: Review of the Phase II and III trials. Clin. Investig..

[30] Branquinho R.T., Roy J., Farah C., Garcia G.M., Aimond F., Le Guennec J.-Y., Saude-Guimarães D.A., Grabe-Guimaraes A., Mosqueira V.C.F., de Lana M. (2017). Biodegradable Polymeric Nanocapsules Prevent Cardiotoxicity of Anti-Trypanosomal Lychnopholide. Sci. Rep..

[31] Branquinho R.T., Pound-Lana G., Marques Milagre M., Saúde-Guimarães D.A., Vilela J.M.C., Spangler Andrade M., de Lana M., Mosqueira V.C.F. (2017). Increased Body Exposure to New Anti-Trypanosomal Through Nanoencapsulation. Sci. Rep..

[32] Branquinho R.T., de Mello C.G.C., Oliveira M.T., Reis L.E.S., de Vieira P.M.A., Saúde-Guimarães D.A., Mosqueira V.C.F., de Lana M. (2020). Lychnopholide in Poly(d,l-Lactide)- block -Polyethylene Glycol Nanocapsules Cures Infection with a Drug-Resistant *Trypanosoma cruzi* Strain at Acute and Chronic Phases. Antimicrob. Agents Chemother..

[33] Mosqueira V.C.F., Mazzeti A.L., Bahia M.T., Formiga F.R., Inamuddin, Severino P. (2021). Chapter 9—Nanomedicines against Chagas disease. Applications of Nanobiotechnology for Neglected Tropical Diseases.

[34] Siqueira R.P., Milagre M.M., de Oliveira M.A., Branquinho R.T., Torchelsen F.K.V., de Lana M., Machado M.G.C., Andrade M.S., Bahia M.T., Mosqueira V.C.F. (2022). In vitro interaction of polyethylene glycol-*block*-poly(*D,L*-lactide) nanocapsule devices with host cardiomyoblasts and *Trypanosoma cruzi*-infective forms. Parasitol. Res..

[35] Okamoto J., Fukunami M., Kioka H. (2007). Frequent premature ventricular contractions induced by itraconazole. Circ. J..

[36] Philips J.A., Marty F.M., Stone R.M., Koplan B.A., Katz J.T., Baden L.R. (2007). Torsades de pointes associated with voriconazole use. Transpl. Infect. Dis..

[37] Tholakanahalli V.N., Potti A., Hanley J.F., Merliss A.D. (2001). Fluconazole-induced torsade de pointes. Ann. Pharmacother..

[38] Leite E.A., Grabe-Guimarães A., Guimarães H.N., Machado-Coelho G.L.L., Barratt G., Mosqueira V.C.F. (2007). Cardiotoxicity reduction induced by halofantrine entrapped in nanocapsule devices. Life Sci..

[39] Moreira Souza A.C., Grabe-Guimarães A., Cruz J.D.S., Santos-Miranda A., Farah C., Teixeira Oliveira L., Lucas A., Aimond F., Sicard P., Mosqueira V.C.F. (2020). Mechanisms of artemether toxicity on single cardiomyocytes and protective effect of nanoencapsulation. Br. J. Pharmacol..

[40] de Sousa D.R.T., de Oliveira Guerra J.A., Ortiz J.V., do Nascimento Couceiro K., da Silva e Silva M.R.H., Jorge Brandão A.R., Guevara E., Arcanjo A.R.L., de Oliveira Júnior E.F., Smith-Doria S. (2023). Acute Chagas disease associated with ingestion of contaminated food in Brazilian western Amazon. Trop. Med. Int. Health.

[41] Fessi H., Puisieux F., Devissaguet J.P., Ammoury N., Benita S. (1989). Nanocapsule formation by interfacial polymer deposition following solvent displacement. Int. J. Pharm..

[42] ANVISA, National Health Surveillance Agency Ministry of Health, BRAZIL (2017). Resolution RDC n° 166, of July 24, 2017. Provides Criteria for the Validation of Analytical Methods. Official Gazette of the Federative Republic of Brazil, Brasília, DF, Section 1 25 July. https://bvsms.saude.gov.br/bvs/saudelegis/anvisa/2017/rdc0166_24_07_2017.pdf.

[43] Santos-Magalhaes N.S., Fessi H., Puisieux F., Benita S., Seiller M. (1995). In vitro release kinetic examination and comparative evaluation between submicron emulsion and polylactic acid nanocapsules of clofibride. J. Microencapsul..

[44] Zingales B., Andrade S., Briones M., Campbell D., Chiari E., Fernandes O., Guhl F., Lages-Silva E., Macedo A., Ma-chado C. (2009). A New Consensus for *Trypanosoma cruzi* Intraspecific Nomenclature: Second Revision Meeting Recommends TcI to TcVI. Mem. Inst. Oswaldo Cruz.

[45] Zingales B., Miles M.A., Moraes C.B., Luquetti A., Guhl F., Schijman A.G., Ribeiro I. (2014). Drug discovery for chagas disease should consider *Trypanosoma cruzi* strain diversity. Mem. Inst. Oswaldo Cruz.

[46] Filardi L.S., Brener Z. (1987). Susceptibility and natural resistance of *Trypanosoma cruzi* strains to drugs used clinically in Chagas’ disease. Trans. R. Soc. Trop. Med. Hyg..

[47] Brener Z. (1962). Therapeutic activity and criterion of cure in mice experimentally infected with *Trypanosoma cruzi*. Rev. Inst. Med. Trop. São Paulo.

[48] Romanha A.J., Castro S.L., De Soeiro M.D.N.C., Lannes-Vieira J., Ribeiro I., Talvani A., Bourdin B., Blum B., Olivieri B., Zani C. (2010). In vitro and in vivo experimental models for drug screening and development for Chagas disease. Mem. Inst. Oswaldo Cruz.

[49] Chiari E., Dias J.C.P., Lana M., Chiari C.A. (1989). Hemocultures for the parasitological diagnosis of human chronic Chagas’ disease. Rev. Soc. Bras. Med. Trop..

[50] Leite E.A., Vilela J.M.C., Mosqueira V.C.F., Andrade M.S. (2005). Poly-Caprolactone Nanocapsules Morphological Features by Atomic Force Microscopy. Microsc. Microanal..

[51] Veloso V.M., Carneiro C.M., Toledo M.J.O., Lana M., Chiari E., Tafuri W.L., Bahia M.T. (2001). Variation in Susceptibility to Benznidazole in Isolates Derived from *Trypanosoma cruzi* Parental Strains. Mem. Inst. Oswaldo Cruz.

[52] Cao X., Sun Z., Cao Y., Wang R., Cai T., Chu W., Hu W., Yang Y. (2014). Design, Synthesis, and Structure-Activity Relationship Studies of Novel Fused Heterocycles-Linked Triazoles with Good Activity and Water Solubility. J. Med. Chem..

[53] Torrico F., Gascon J., Ortiz L., Alonso-Vega C., Pinazo M.-J., Schijman A., Almeida I.C., Alves F., Strub-Wourgaft N., Ribeiro I. (2018). E1224 Study Group. Treatment of adult chronic indeterminate Chagas disease with benznidazole and three E1224 dosing regimens: A proof-of-concept, randomised, placebo-controlled trial. Lancet Infect. Dis..

[54] Bartroli J., Turmo E., Algueró M., Boncompte E., Vericat M.L., Conte L., Ramis J., García-Rafanell J., Forn J. UR-9825: A New Triazole Derivative with Potent, Broad-Spectrum Antifungal Activity. Program and Abstracts of the 37th Interscience Conference on Antimicrobial Agents and Chemotherapy (ICAAC), 1997, abstr. E-67, September, Toronto, Canada. American Soc. for Microbiology, Washington, DC. https://search.worldcat.org/pt/title/633698851.

[55] Trindade I.C., Pound-Lana G., Perera D.G.S., De Oliveira L.A.M., Andrade M.S., Vilela J.M.C., Postacchini B.B., Mosqueira V.C.F. (2018). Mechanisms of interaction of biodegradable polyester nanocapsules with non-phagocytic cells. Eur. J. Pharm. Sci..

[56] Chen C.K., Leung S.S., Guilbert C., Jacobson M.P., McKerrow J.H., Podust L.M. (2010). Structural Characterization of CYP51 from *Trypanosoma cruzi* and *Trypanosoma brucei* Bound to the Antifungal Drugs Posaconazole and Fluconazole. PLoS Negl. Trop. Dis..

[57] Dumoulin P., Vollrath J., Tomko S.S., Burleigh B. (2020). Glutamine metabolism modulates azole susceptibility in *Trypanosoma cruzi* amastigotes. eLife.

[58] Souza A.C.M., Mosqueira V.C.F., Silveira A.P.A., Antunes L.R., Richard S., Guimarães H.N., Grabe-Guimarães A. (2018). Reduced cardiotoxicity and increased oral efficacy of artemether polymeric nanocapsules in *Plasmodium berghei*-infected mice. Parasitology.

[59] Mosqueira V.C.F., Loiseau P.M., Bories C., Legrand P., Devissaguet J.-P., Barratt G. (2004). Efficacy and Pharmacokinetics of Intravenous Nanocapsule Formulations of Halofantrine in *Plasmodium berghei*-Infected Mice. Antimicrob. Agents Chemother..

[60] Mosqueira V.C.F., Legrand P., Barratt G. (2006). Surface-Modified and Conventional Nanocapsules as Novel Formulations for Parenteral Delivery of Halofantrine. J. Nanosci. Nanotechnol..

[61] Tarlenton R.L. (2001). Parasite persistence in the aetiology of Chagas disease. Int. J. Parasitol..

