# Supplementary material for: Albaconazole Polymeric Nanocapsules for Treating Trypanosoma cruzi Infections
Source: Pathogens. 2025 Mar 26;14(4):319. doi: 10.3390/pathogens14040319 (PMC12030734; doi:10.3390/pathogens14040319)
Supplement: Supplementary file 1 [file pathogens-14-00319-s001.zip › pathogens-3485749-supplementary.pdf]

Supplementary material:

ALBACONAZOLE POLYMERIC NANOCAPSULES FOR TREATING *Trypanosoma cruzi* INFECTIONS

Cristina Maria de Barros[1,2], Vanja Maria Veloso[3], Margareth Spangler Andrade[4], José Mário Carneiro Vilela[4], Maria Alice de Oliveira[1], Marta de Lana[2,5], Maria Terezinha Bahia[2,6] and Vanessa Carla Furtado Mosqueira[1,2,3]*

S1-Development of an analytical methodology using ultraviolet spectrometry for measuring albaconazole

S1.1. ABZ Spectrum

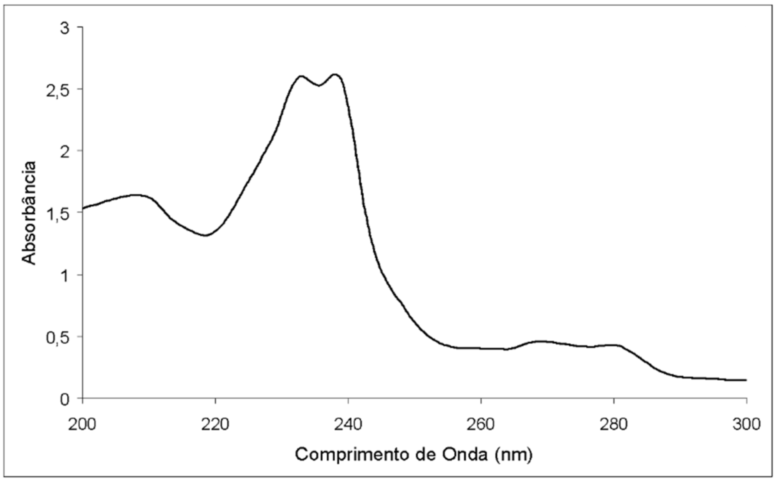

Figure S1: Ultraviolet spectrum showing the maximum absorption peak of ABZ in acetonitrile (5 mg/mL) at 238 nm.

S1.2. Linearity

The standard curve of ABZ in acetonitrile, constructed from the average absorbances at concentrations of 0.25, 0.5, 1, 2.5, 5, 7.5, 10, and 25 µg/mL, is represented in Figure S2, along with the straight-line equation and the coefficient of determination (r^2).

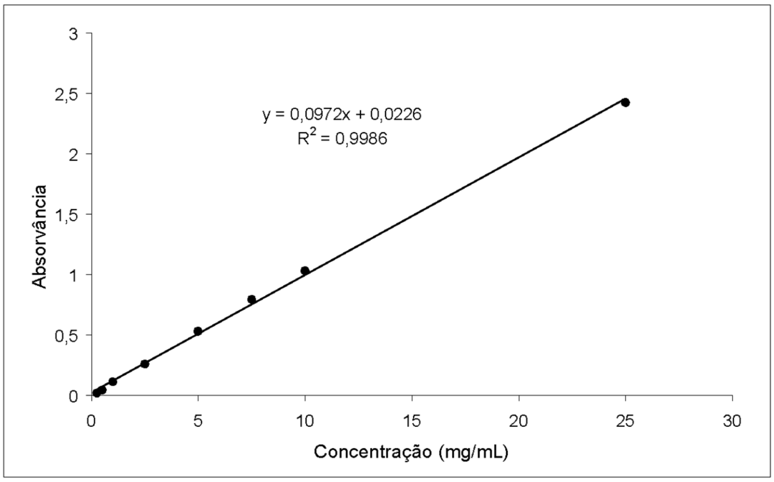

Figure S2: ABZ calibration curve in acetonitrile at a wavelength of 238 nm; R^2 = coefficient of determination.

The value of r^2 found means that 99.86% of the data is explained by the curve. The calibration curve presented a correlation coefficient value of r = 0.9986, demonstrating the existence of a linear correlation between concentrations and absorbances, in the range of 0.25 to 25 µg/mL. The equation representing linearity was y = 0.0972x + 0.0226, where the value associated with the variable x corresponds to the slope of the line and 0.0226 to the intercept of the line with the ordinate axis. The average absorbance values at 238 nm of the different concentrations of ABZ in acetonitrile are represented in Table 3. According to the ANVISA Analytical and Bioanalytical Methods Validation Guide (ANVISA 2003), the results show that there was linearity, as the value of CV is below 5%.

Table S1: ABZ average absorbance values as a function of its concentration

ABZ (µg/mL)	Medium Absorbance (at 238 nm)[a] ± DP[1]	CV[2] (%)
0,25	0,018 ± 0,0001	0,55
0,5	0,043 ± 0,0002	0,46
1,0	0,113 ± 0,0001	0,09
2,5	0,259 ± 0,0005	0,19
5,0	0,531 ± 0,0004	0,07
7,5	0,792 ± 0,0009	0,11
10,0	1,032 ± 0,0007	0,07
25,0	2,422 ± 0,0007	0,03

[a] n = 3; [1]SD = standard deviation; [2]CV = coefficient of variation, given by :(SD/average absorbance) x 100.

S1.3. Specificity

The interference of other components of the nanostructured system in reading ABZ absorbances was evaluated when 1 or 10% white NC samples were used as blanks. The fact that the coefficient of variation (CV) is less than 5%, as shown in Table 2, guarantees that at a wavelength of 238 nm, the absorbance of the drug does not suffer interference from other NC constituents at concentrations of 1% and 10 %.

Table S2: Results of the calibration curve made with pure ABZ, ABZ + NC at 1% and ABZ + NC at 10%.

ABZ (µg/mL)	Medium Absorbance (at 238 nm)[a] ± DP[1]	CV[2](%)
0,25	0,017 ± 0,0008	4,67
0,5	0,042 ± 0,0008	1,89
1,0	0,109 ± 0,0047	4,28
2,5	0,262 ± 0,0044	1,67
5,0	0,538 ± 0,0104	1,93
7,5	0,79 ± 0,0028	0,35
10,0	1,029 ± 0,0031	0,3
25,0	2,427 ± 0,0073	0,3

[a] n = 3; [1]SD = standard deviation; [2]CV = coefficient of variation, given by :(SD/average absorbance) x 100.

S1.4. Precision

Repeatability (intra-run precision)

Table 3 shows the absorbance values corresponding to samples of concentrations 1, 5, and 10 µg ABZ/mL, obtained in triplicate and on the same day, and CV of each one.

Table S3: Results of the repeatability test to validate the spectrophotometric assay of ABZ at 238 nm.

Theoretical concentration (µg/mL)	Average absorbance ± DP[1]	CV[2] (%)
1	0,113 ± 0,0001	0,0885
5	0,531 ± 0,0005	0,0942
10	1,032 ± 0,0007	0,0678

[a] n = 3; [1]SD = standard deviation; [2]CV = coefficient of variation, given by :(SD/average absorbance) x 100.

The method presented CV in the range of 0.0678 to 0.0942% for all concentrations analyzed, with values below 5% in accordance with the acceptance criteria established for precision in the Validation Guide for Analytical and Bioanalytical Methods from ANVISA (ANVISA, 2003). These values demonstrate the good precision of the method in terms of repeatability.

Intermediate accuracy

Intermediate precision was evaluated by calculating the CV of absorbance values, obtained in triplicate, on two different days for concentrations of 1, 5, and 10 µg ABZ/mL. The results are found in Table 4.

The results showed that the coefficients of variation obtained in the analyzes between days were less than 5%, and it can be concluded that the method is reproducible, without showing significant variations between days.

Table S4: Results of the intermediate precision test, in two days, to validate the spectrophotometric measurement of ABZ at 238 nm.

Theoretical concentration (µg/mL)	Average absorbance[a] ± DP[1]	CV[2] (%)
1	0,1092 ± 0,0054	4,94
5	0,5416 ± 0,0149	2,75
10	1,0408 ± 0,0126	1,21

[a] n = 3; [1]SD = standard deviation; CV = coefficient of variation, given by :(SD/average absorbance) x 100.

S1.5. Accuracy

Accuracy was assessed by comparing concentration values obtained in triplicate from absorbance readings of concentrations of 1, 5, and 10 mg ABZ/mL with their theoretical values as shown in Table 5.

The results showed that the values obtained for accuracy varied from a minimum value of 93% for concentration 1 mg/mL, up to a maximum value of 104.6%, for concentration 5 mg/mL, with the average value being 100.48%. These data show that the values found in the experiments differed by a maximum of around 4.8% from the theoretical values, indicating good accuracy for the spectrophotometric dosage of ABZ.

Table S5: Accuracy of the ABZ determination method.

Theoretical concentration (µg/mL)	Absorbance ± DP[1]	Experimental concentration (µg/mL)	Accuracy (%)
1	0,113 ± 0,0001	0,9300	93
5	0,531 ± 0,0005	5,2305	104,6
10	1,032 ± 0,0007	10,3837	103,84

[a] n = 3; [1]SD = standard deviation.

Validation of the analytical methodology for ABZ measurement was carried out using UV spectrophotometry. The developed method showed linearity in the range of 0.25 to 25 mg/mL, precision and accuracy of the data obtained. The intra- and inter-day CVs of method calibration were low and within the maximum established limits. The method was therefore considered validated for determining the drug in the samples to be analyzed.

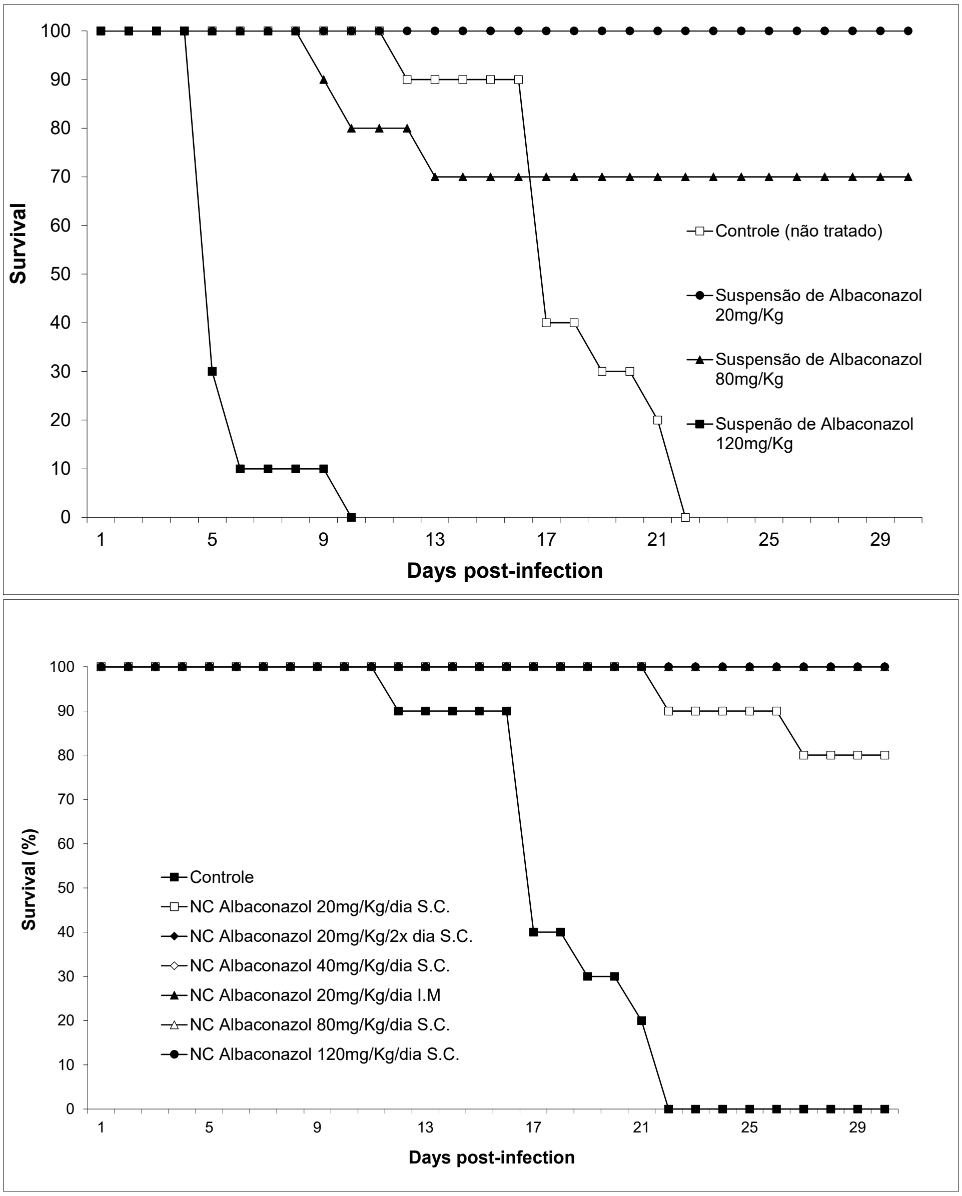

Figure S3: Survival after treatment with different doses of Albaconazole coarse suspensions and ABZ-Nanocapsules.

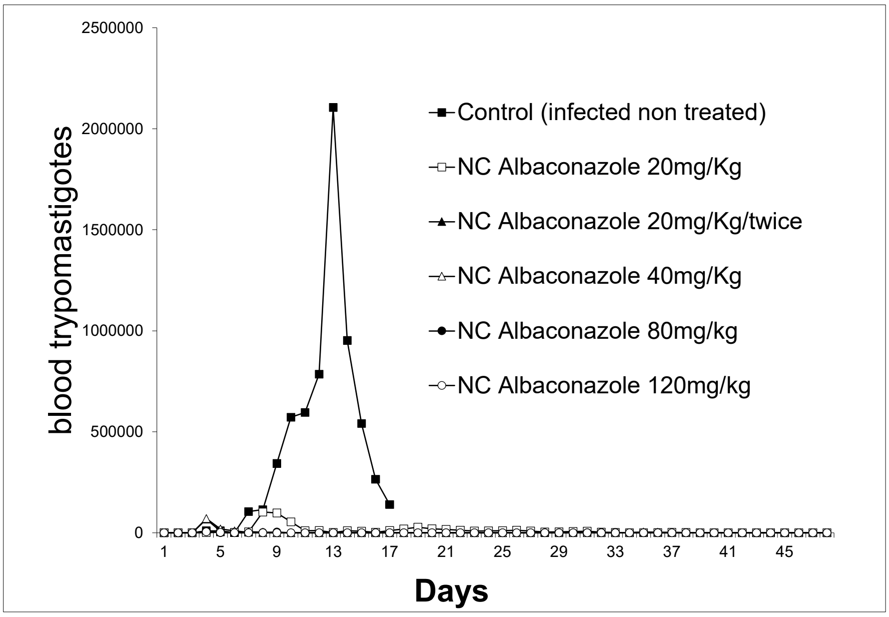

Figure S4: Parasitemia curves after treatment with different doses of Albaconazole loaded in NCs.
